# A Host-Restricted Self-Attenuated Influenza Virus Provides Broad Pan-Influenza A Protection in a Mouse Model

**DOI:** 10.3389/fimmu.2021.779223

**Published:** 2021-12-02

**Authors:** Minjin Kim, Yucheol Cheong, Jinhee Lee, Jongkwan Lim, Sanguine Byun, Yo Han Jang, Baik Lin Seong

**Affiliations:** ^1^ Graduate Program in Biomaterials Science and Engineering, College of Life Science and Biotechnology, Yonsei University, Seoul, South Korea; ^2^ Department of Biotechnology, College of Life Science and Biotechnology, Yonsei University, Seoul, South Korea; ^3^ Department of Integrated OMICS for Biomedical Science, College of Life science and Biotechnology, Yonsei University, Seoul, South Korea; ^4^ Department of Biological Sciences and Biotechnology Major in Bio-Vaccine Engineering, Andong National University, Andong, South Korea; ^5^ Vaccine Industry Research Institute, Andong National University, Andong, South Korea; ^6^ Department of Microbiology, College of Medicine, Yonsei University, Seoul, South Korea; ^7^ Vaccine Innovative Technology ALliance (VITAL)-Korea, Yonsei University, Seoul, South Korea

**Keywords:** influenza virus, universal vaccine, host-restriction, live-attenuated vaccine, cross-protection

## Abstract

Influenza virus infections can cause a broad range of symptoms, form mild respiratory problems to severe and fatal complications. While influenza virus poses a global health threat, the frequent antigenic change often significantly compromises the protective efficacy of seasonal vaccines, further increasing the vulnerability to viral infection. Therefore, it is in great need to employ strategies for the development of universal influenza vaccines (UIVs) which can elicit broad protection against diverse influenza viruses. Using a mouse infection model, we examined the breadth of protection of the caspase-triggered live attenuated influenza vaccine (ctLAIV), which was self-attenuated by the host caspase-dependent cleavage of internal viral proteins. A single vaccination in mice induced a broad reactive antibody response against four different influenza viruses, H1 and rH5 (HA group 1) and H3 and rH7 subtypes (HA group 2). Notably, despite the lack of detectable neutralizing antibodies, the vaccination provided heterosubtypic protection against the lethal challenge with the viruses. Sterile protection was confirmed by the complete absence of viral titers in the lungs and nasal turbinates after the challenge. Antibody-dependent cellular cytotoxicity (ADCC) activities of non-neutralizing antibodies contributed to cross-protection. The cross-protection remained robust even after *in vivo* depletion of T cells or NK cells, reflecting the strength and breadth of the antibody-dependent effector function. The robust mucosal secretion of sIgA reflects an additional level of cross-protection. Our data show that the host-restricted designer vaccine serves an option for developing a UIV, providing pan-influenza A protection against both group 1 and 2 influenza viruses. The present results of potency and breadth of protection from wild type and reassortant viruses addressed in the mouse model by single immunization merits further confirmation and validation, preferably in clinically relevant ferret models with wild type challenges.

## Introduction

Influenza is a highly contagious respiratory disease that presents a considerable burden to public health and the economy worldwide. Approximately three to five million people are infected with influenza viruses annually, and approximately 290,000 to 650,000 people die due to influenza (1). Influenza viruses show considerable genetic diversity with various subtypes depending on the combination of surface hemagglutinin (HA) and/or neuraminidase (NA) genes (2, 3). Additionally, influenza viruses, which possess segmented RNA genomes, demonstrate a high level of genetic variability, resulting in frequent antigenic changes through antigenic drift and shift (4, 5). Due to the antigenic diversity and variability of influenza viruses, close monitoring and surveillance of influenza virus strains are required to produce effective influenza vaccines, which closely match the antigenicity of the circulating strains. The World Health Organization (WHO) annually updates its recommendations for the vaccine strains to target the viruses predicted to be the most frequently circulating in the coming season (6). Currently, licensed influenza vaccines primarily depend on HA-specific neutralizing antibodies, thus providing a very narrow strain-specific protection. Therefore, even a slight mismatch between the vaccine and target virus may significantly compromise the protective efficacy of the vaccine, leading to a significant reduction in vaccine efficacy (7). Additionally, the possibility of the emergence of a new influenza virus that causes a global pandemic through gene exchange between different types cannot be excluded (3, 8). Furthermore, human infection caused by highly pathogenic avian influenza viruses, such as H5, H7, and H9 strains, can cause high mortality in infected individuals (8, 9). Therefore, the development of a universal influenza vaccine (UIV) that can protect against various influenza viruses is highly desirable (10–12).

To overcome these concerns, vaccines should elicit “universal” protective immune responses against influenza viruses. UIVs have been developed using several strategies to induce cross-reactive immune responses. Classical seasonal vaccines usually elicit neutralizing antibodies against the globular head of HA, where the receptor-binding domain (RBD) is located. However, the globular domain is too variable across subtypes to elicit broad immune protection (13, 14). Therefore, UIVs that target the conserved regions of influenza surface proteins, such as HA stalk domain or M2 ectodomain (M2e), have been developed, with notable achievements in cross-reactive immune responses, especially for the HA stalk (15–17). Various strategies of UIV targeting conserved regions have been documented, including recombinant proteins, nucleic acid-based (DNA/RNA) vaccines, or in combination with live attenuated vaccines (13, 18). However, these strategies have limitations that only partial protection within the same HA group is achieved, rather than pan-influenza A protection covering group 1 and 2 viruses (19). Furthermore, compared to live attenuated influenza vaccines (LAIVs) that induce both mucosal and humoral immune responses, recombinant protein vaccines are usually unable to elicit potent cross-protective mucosal immunity (15, 20). Currently, used LAIVs are cold-adapted LAIVs (CAIVs). However, despite their potent protection efficacy, the development of CAIVs as UIVs is limited by possible genetic mutations in the RNA genome and a decrease in production titer by the attenuated phenotype (21, 22). Thus, the rational design of clinically useful LAVs is still challenging, and deploying LAIVs as a UIV remains highly empirical.

Apoptosis and caspase activation are integral components of general host antiviral responses induced by viral infection (23, 24). By investigating these tightly regulated host defense mechanisms, we previously presented a novel apoptosis-triggered attenuation of viral virulence as a rational design of ctLAIVs (25). Despite significant attenuation, the viruses demonstrated a high-growth phenotype in embryonated eggs at low temperatures, ensuring their productivity. In the present study, the designer virus was evaluated for cross-reactive immune responses against influenza A viruses, including group1 (H1N1 and H5N1) and group2 (H3N2 and H7N1). Using a mouse model, we confirmed pan-influenza A protection with complete viral clearance in the respiratory tract after vaccination. Antibody-dependent cellular cytotoxicity (ADCC) has been proposed as a major mode of action for heterosubtypic protection (26). These promising results could guide the development of a mechanism-based LAIV as a potential UIV candidate with a desirable level of potency, breadth, and safety.

## Materials and Methods

### Ethical Statement

All experiments were performed in accordance with the guidelines of the Ministry of Food and Drug Safety (MFDS) of the Korean Government. All experimental procedures were conducted with the approval of the Institutional Biosafety Committee of Yonsei University (Permit number: IBC-A-202012-264-01), and animal experiments were performed with the approval of the Institutional Animal Care and Use Committee (IACUC) of the International Vaccine Institute (IVI) and strict follow-up management (Permit number: IACUC PN 2021-001).

### Cell Line and Viruses

The LAIV used in this study is a genetically modified influenza A virus A/Puerto Rico/8/34 (H1N1), a mechanism-based self-attenuated virus that inserts a caspase cleavage motif into the *NP* and *NS* genes (25). The cross-protective efficacy was evaluated by four different influenza A viruses (IAVs): A/Puerto Rico/8/34 (PR8, H1N1), A/Philippines/2/82 (Phil82, H3N2), 7:1 single gene reassortant virus PR8: HA of A/Indonesia/5/05 (reIndo05, H5N1), and PR8: HA of A/Netherlands/219/03 (reNet03, H7N1) (27). The mouse lethal dose (mLD_50_) was determined by a preliminary experiment, 1×10^3^ PFU (H1N1), 2.5×10^2^ PFU (H3N2), 3×10^5^ PFU (H5N1), and 2.5×10^4^ PFU (H7N1) respectively. Madin-Darby canine kidney (MDCK) cells were cultured in minimum essential medium (MEM) (Hyclone Laboratories, US) supplemented with 10% fetal bovine serum (FBS, Hyclone Laboratories, US).

### Vaccination and Challenge

Six-week-old female BALB/c mice were supplied by Orient Bio Inc. (Seoul, Korea). After acclimatization for 7 days, mice were inoculated with 50 μL of phosphate-buffered saline (PBS) or 10^5^ plaque-forming unit (PFU)/50 μL of LAIV by intranasal administration following the immunization protocol in previous studies (25). Mice were anesthetized with a 2:2:1 mixture of PBS, Alfaxan (Jurox, Australia), and 5% xylazine. Sera were collected by retro-orbital bleeding under anesthesia on days 35 and 49. On day 56, mice were intranasally challenged with viruses (H1N1, H3N2, H5N1, and H7N1) with 2 mLD_50_ *via *the intranasal route. After challenge, the body weight and survival of mice were monitored daily for two weeks.

### Collection of Mice Tissues for Viral Titration

For quantification of viral replication, mice were sacrificed and tissue samples were collected for viral titration on days 3, 5, and 7 post-infection (dpi) (*N*=5). After anesthesia, whole lungs were removed from mice and were homogenized with 1 mL of PBS. After centrifugation for 20 min at 12,000 rpm, the supernatants were collected. Nasal turbinate samples were collected by lavaging with 200 μL of PBS. All samples were aliquoted and stored at -80°C.

### *In Vivo* Immune Cell Depletion

For depletion of CD4+ T cells, CD8+ T cells, and NK cells *in vivo*, 200 μg of anti-CD4 mAb (clone 2.43; BioXcell, US), anti-CD8 mAb (clone GK1.5; BioXcell, US), and 20 μl of anti-asialo GM1 antiserum (Wako Pure Chemical Industries, Japan), respectively, were intraperitoneally injected into each mouse three times on days 3,5, and 7 before challenge (27). Control mice were injected with 200 μg of rat IgG2b (clone LTF-2, BioXcell, US). The body weight of the mice was measured for two weeks after challenge.

### Influenza HA Proteins

HA proteins, either recombinant or virus-derived, were used for enzyme-linked immunosorbent assay (ELISA). The four HA proteins of influenza A, including A/Puerto Rico/8/34 (H1N1), A/Philippines/2/02 (H3N2), A/Indonesia/5/05 (H5N1), and A/Netherlands/219/03 (H7N7), expressed in insect or mammalian cells, were purchased from Sino Biological Inc. The egg-derived standard HA antigens of H1N1 strains, A/Puerto Rico/8/1934, A/Singapore/Gp1908/2015, A/Brisbane/59/2007, and A/Michigan/45/2015 egg-derived antigens, were purchased from NIBSC. The recombinant globular domain (GD) and HA stalk of A/Puerto Rico/8/34 (27) and consensus HA expressed in *Escherichia coli* were purified and used as coating antigens in ELISA (28).

### ELISA

ELISA was performed to analyze whether the vaccine-induced antibodies specifically bind to the whole influenza virus or protein antigen. Ninety-six-well plates (SPL, Korea) were coated with 100 μL of 10^5^ PFU/well whole viruses or 100 μL of 0.1 μg/well of proteins overnight at 4°C. The plates were washed three times with 120 μL/well of PBST (0.05% Tween20 in PBS, pH 7.5) and blocking with 150 μL/well of blocking buffer (1% [w/v] BSA in PBST) for 1 h at room temperature (RT). Then, the plates were incubated with 100 μL/well of two-fold serial diluted mouse sera or nasal turbinate samples for 1 h at RT. After washing three times, the plates were incubated with 100 μL/well of 1:10,000 or 1:5,000 diluted horse-radish peroxidase (HRP)-conjugated secondary goat anti-mouse IgG1, IgG2a, or IgA (Bethyl, US) for 1 h at RT. Then, the plates were washed and incubated with 100 μL/well TMB substrate solution (BD Biosciences, UK) for 30 min at RT. After color development, 50 μL/well 2N of sulfuric acid (H_2_SO_4_) solutions was added to stop the reaction, and the optical density at 450 nm (OD_450nm_) was measured on an ELISA reader (BMG Labtech, Germany).

### Plaque Assay

To titrate viruses of the lungs and nasal turbinates, MDCK cells in confluent 12-well culture plates were washed with 500 μL/well of PBS and incubated with 200 μL/well of ten-fold serial diluted samples on a rocker for 45 min at RT. After suction removal of the viral solution, cells were washed and added with 1.5 mL/well of overlay consisting of Dulbecco’s Modified Eagle Medium (DMEM) containing 1% (w/v) low melting agarose and 10 μg/mL trypsin. After the overlays were hardened, the plates were incubated in a 5% CO_2_ humidified incubator at 37°C until plaques were formed. To count the plaques, the cells were treated with 4% formaldehyde and stained with crystal violet solution.

### Plaque Reduction Neutralization Test

To evaluate the neutralization ability, a PRNT was performed using pooled mouse sera. Sera were diluted in MEM (1:25 dilution) and heat-inactivated at 56°C for 30 min. Two-fold serially diluted sera were mixed with 110 PFU of viruses in a 1:1 ratio and then incubated at 37°C for 90 min. The mixtures were added to MDCK monolayers grown in 12-well cell culture plates (SPL, Korea) and incubated at RT for 45 min. The plaque assay was performed as described previously.

### ADCC Assay

The ADCC assay was performed using the mFcγRIV ADCC Reporter Bioassay Kit (Promega, US) following the manufacturer’s instructions. In brief, confluent MDCK cells grown in white 96-well plates (SPL, Korea) were infected with influenza viruses (multiplicity of infection of 1) diluted in MEM and incubated in MEM at 37°C. The next day, the supernatants were suction-removed and 100 μL of sera (1:50 dilution) and mFcγRIV effector cells were added to the MDCK cells, and the cells were incubated at 37°C for 6 h. Then, Bio-Glu™ reagent was added to each well for expression of luminescence. A luminescence plate reader (BMG Labtech, Germany) was used to detect ADCC activity.

### Statistical Analysis

All values are expressed as the mean ± standard deviation (SD) of each cohort. To compare the differences between the two groups, an unpaired, two-tailed *t*-test was performed, and to compare the differences among the three groups, one-way analysis of variance (ANOVA) was performed. Statistical significance was set at <0.05 (***P < 0.001; **P < 0.01; *P < 0.05).

## Results

### Cross-Protection Against Heterosubtypic Influenza A Viruses in Mice

**Figure 1:**
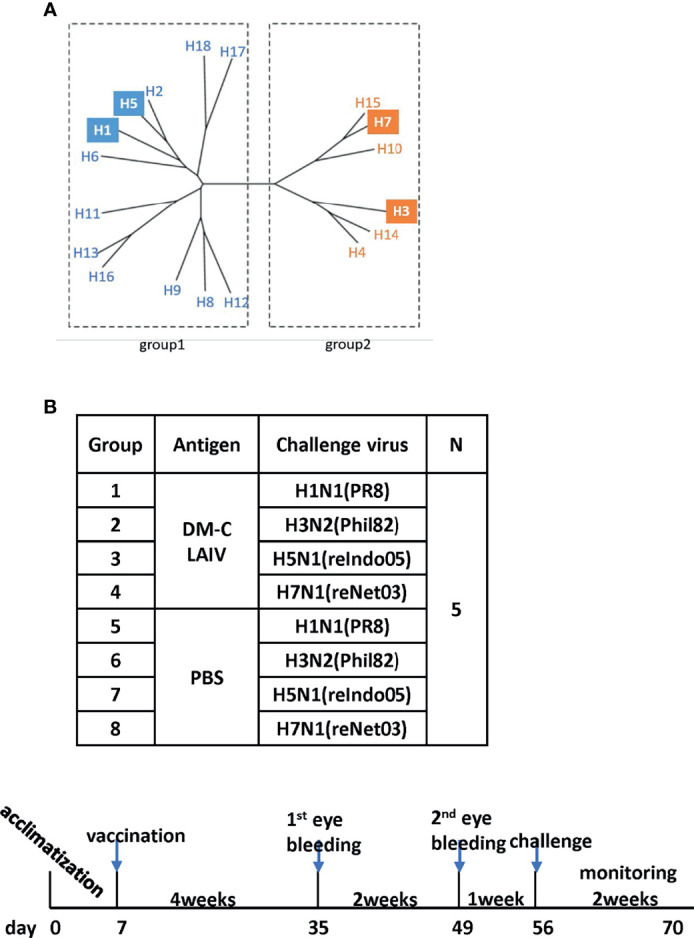
Examining of cross-protection against four types of influenza A viruses in an animal model. **(A)** Phylogenetic tree of the influenza A virus. Influenza A virus has a total of 16 HA subtypes and is divided into groups 1 and 2. As indicated by color boxes, four HA subtypes were used for *in vivo* experiments and antibody analysis. **(B)** Schematic diagram of the schedule of immunogenicity and collection of mice sera. Mice sera were collected at 4 and 6 weeks after vaccination and were challenged with four types of influenza A viruses.

**Figure 2:**
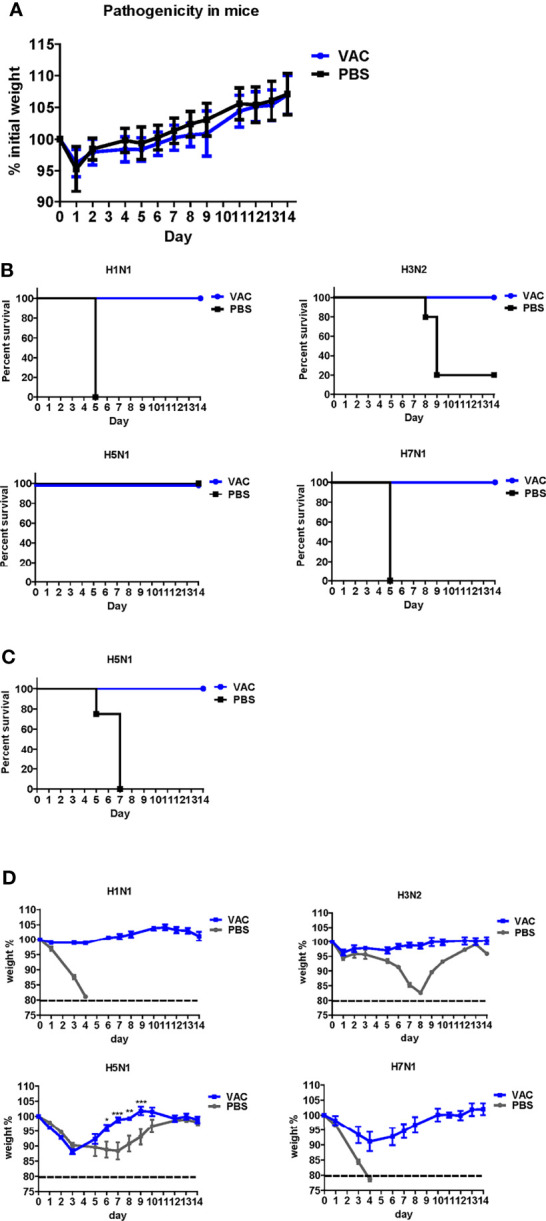
Protective efficacy of DM-C against four types of influenza A viruses. **(A)** Pathogenicity confirmation of DM-C in animal models. After acclimating six-week-old mice for 1 week, the mice were vaccinated with 10^5^ PFU/50 μL of DM-C LAIV (group VAC) or 50 μL of PBS (PBS group). The mice were observed daily for 2 weeks to check changes in the body weight and survival rate. No clinical symptoms were observed in both groups. The graph shows the mean of each cohort (*N=20*), and the error bar means the standard deviation (SD). **(B, C)** Mice were challenged with two subtypes of group 1 and 2 influenza A viruses each. Mice were challenged with H1N1, H3N2, and H7N1 at 2 mLD50 and with H5N1 (6×10^5^ PFU) at a survivable dose. Mice were monitored daily. The survival rates **(B)** and changes in bodyweight **(D)** are shown. The humane endpoint is a weight-loss reduction of more than 20%, which is indicated by a dotted line in the graph. The graph shows the mean of each cohort *(N=5)*, and the error bar means the SD. Two-way *ANOVA* with RM was performed for comparing the differences between body weight of VAC group and PBS group (***P < 0.001; **P < 0.01; *P < 0.05). **(C)** Mice were challenged with a lethal dose of H5N1 (2×10^6^ PFU) and were monitored daily for body weight and mortality. The survival rates are shown *(N=4)*.

The backbone of the ctLAIV used in this study was A/Puerto Rico/8/34 (PR8, H1N1), in which the caspase cleavage sequence was introduced into two internal genes, *NP* and *NS1* (double mutants with caspase-sensitivity; DM-C) (25). The attenuation properties of DM-C strain were previously characterized in terms of optimal growth temperature, virulence in mice, and the levels of proinflammatory cytokines induced by vaccination (25). To evaluate the breadth of cross-protection, two different HA groups of influenza virus were included as challenge strains (**Figure 1A**), including homologous PR8 (H1) and heterosubtypic rH5N1 from group 1 and heterosubtypic H3N2 and rH7N1 from group 2 viruses. Before the challenge, we confirmed the pathogenicity of DM-C in a mouse model. Compared to the PBS-treated group, the DM-C vaccinated group did not show appreciable signs of virulence, confirming the attenuation of virulence (**Figure 2A**). The experiment was designed to evaluate the spectrum of protection conferred by DM-C. The mice were challenged seven weeks after a single vaccination and were monitored for their body weight and survival rates for two weeks (**Figure 1B**). The DM-C vaccination provided protection against all four influenza viruses (**Figures 2B, D**). Incidentally, the challenge with H5N1 (6×10^5^ PFU) was not 100% lethal in the experimental setting, but protection by vaccination was apparent by significant improvements in body weight. An additional challenge study with a lethal dose confirmed full protection from the H5N1 heterosubtypic challenge (2×10^6^ PFU) (**Figure 2C** **)**. The results confirmed and further extended heterosubtypic protection from a lethal challenge from a mouse-adapted H5N2 strain (25).

### Protection in the Respiratory Tract

**Figure 3:**
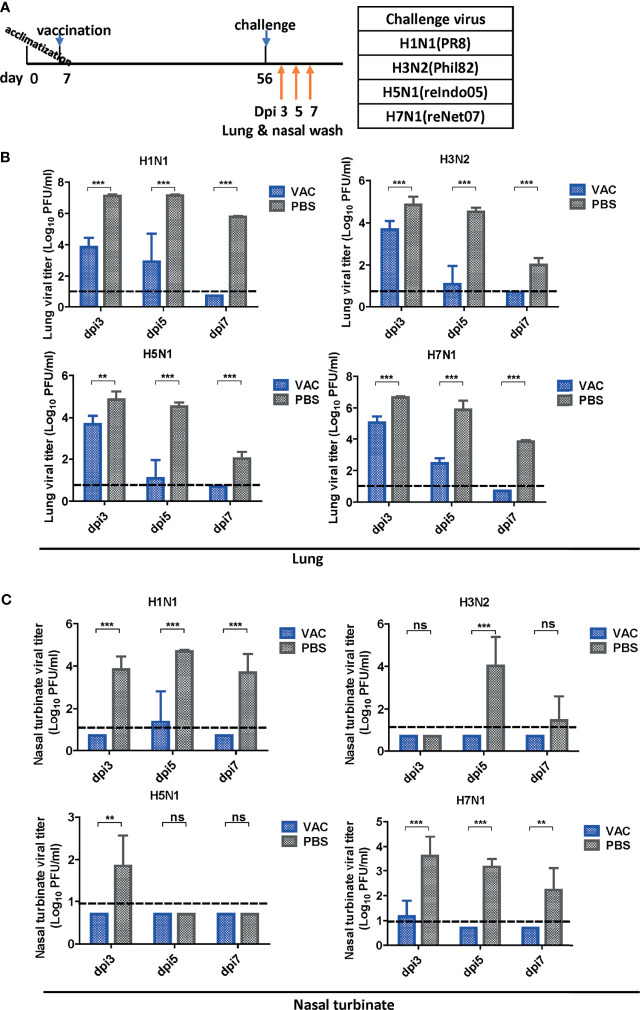
Viral clearance in the respiratory tract after challenge. **(A)** Schematic diagram of the schedule of harvesting lung and nasal turbinate samples. Lungs and turbinate samples were harvested on days 3, 5, and 7 post-infection (dpi) to test the sterile immunity after challenge with four types of viruses. **(B, C)** Sterile immunity in the mouse lungs and turbinate samples against four types of influenza A viruses challenge. The mice vaccinated with DM-C LAIV or PBS were sacrificed at 3, 5, and 7 dpi for collection of lungs **(B)** and turbinate samples **(C)** to titrate viruses by plaque assay. The detection limit was indicated by a dotted line in the graph. The graph shows the mean of each cohort *(N=5)*, and the error bar means the standard deviation (SD). Two-tailed *t-*test was performed for comparing the differences between DM-C LAIV-vaccinated and PBS-treated groups (***P < 0.001; **P < 0.01; ns means not significant).

After vaccination and challenge, the lungs and nasal turbinates of challenged mice were obtained on days 3, 5, and 7 dpi (**Figure 3A**). Virus replication was significantly reduced in the DM-C-vaccinated group compared to that in the PBS group (**Figure 3B**). As early as 3 dpi, there was a 1–2 log (H3, H5, and H7) or more (>3 log; 1,000-fold reduction) (H1) reduction in the viral load. The reduction was greater at 5 dpi, with a 3 log difference in H3, H5, and H7 groups, and >4 log difference in the H1 group. The greater reduction in the H1N1 group reflects superior protection from the homologous H1N1 (PR8) challenge. Remarkably, in all DM-C vaccinated groups, the virus titer was reduced below the detection limit at 7 dpi, in clear contrast to the PBS control, where virulence was still apparent, ranging from 10^2^ PFU (H5) to 5 x 10^5^ PFU (H1). All challenge studies were conducted in a BSL2 type facility, and therefore, the experimental design was limited by the use of reassortant viruses H5N1 (reIndo05) and H7N1 (reNet03) rather than wild type viruses (requiring a BSL3 facility). Challenge virus titers were also estimated in the nasal turbinates (**Figure 3C**). For all viruses, DM-C vaccination greatly reduced the viral load throughout the post-infection period. Again, the most dramatic reduction (1,000–10,000 fold) was observed with the H1 homologous challenge, and robust reduction was also noted with the heterologous challenge (100 fold for H5; 100~1,000 fold for H3 and H7). The smaller effect observed with the H5 (group 1) than that of H3 and H7 (group 2) was probably due to the sub-lethal dose of H5 challenge (**Figures 1B, C**). Thus, the viral load was effectively inhibited in the respiratory tracts of DM-C-vaccinated mice, suggesting a potent sterile immunity conferred by DM-C LAIV.

### DM-C Vaccination Elicits Cross-Reactive Antibody Responses

**Figure 4:**
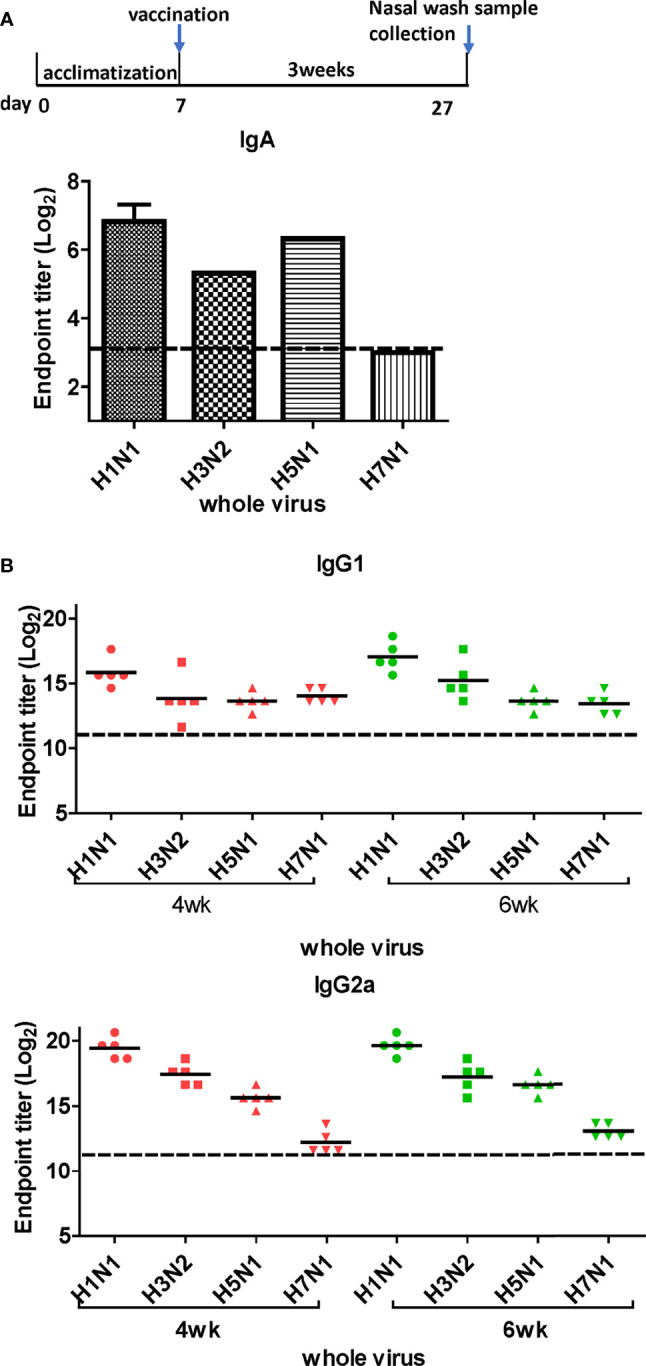
Titration of cross-reactive antibodies by DM-C vaccination. **(A)** Response of IgA antibodies to whole influenza A viruses in the respiratory system. Samples were diluted two-fold serially to measure the titer of IgA antibodies induced by LAIV vaccination in nasal turbinate samples harvested 3 weeks after vaccination. The graph shows the endpoint (E.P) of the pooled sample (*N=2*). **(B)** Response of cross-protective systemic antibodies to whole influenza A viruses. Influenza virus-specific IgG1 and IgG2a were detected by ELISA. Two-fold serial diluted mice sera collected at 4 or 6 weeks post-vaccination were bound to whole viruses. The graph shows the endpoint (E.P) of each cohort *(N=5).* E.P means the reciprocal serum dilution of the vaccinated group that yielded OD_450_ greater than the mean + 2SD of the PBS-treated group.

LAIVs effectively induce mucosal secretory immunoglobulin A (sIgA) antibodies. To examine whether DM-C induces sIgA antibodies, ELISA was performed with the nasal turbinates using H1N1 (PR8), H3N2 (Phil82), H5N1 (reIndo05), and H7N1 (reNet03) viruses as coating antigens (**Figure 4A**). Cross-reactivity was detected against three viruses, except for H7N1. While the highest IgA antibody titer was measured against the homologous H1N1, there was a significant response against H5 (group 1) and H3 (group 2) as well. The response was relatively poor against H7N1, which was noted to have much less immunogenicity than other influenza A viruses (29, 30). Overall, the results suggest that the mucosal immune response contributes greatly to the observed cross-protection.

**Figure 5:**
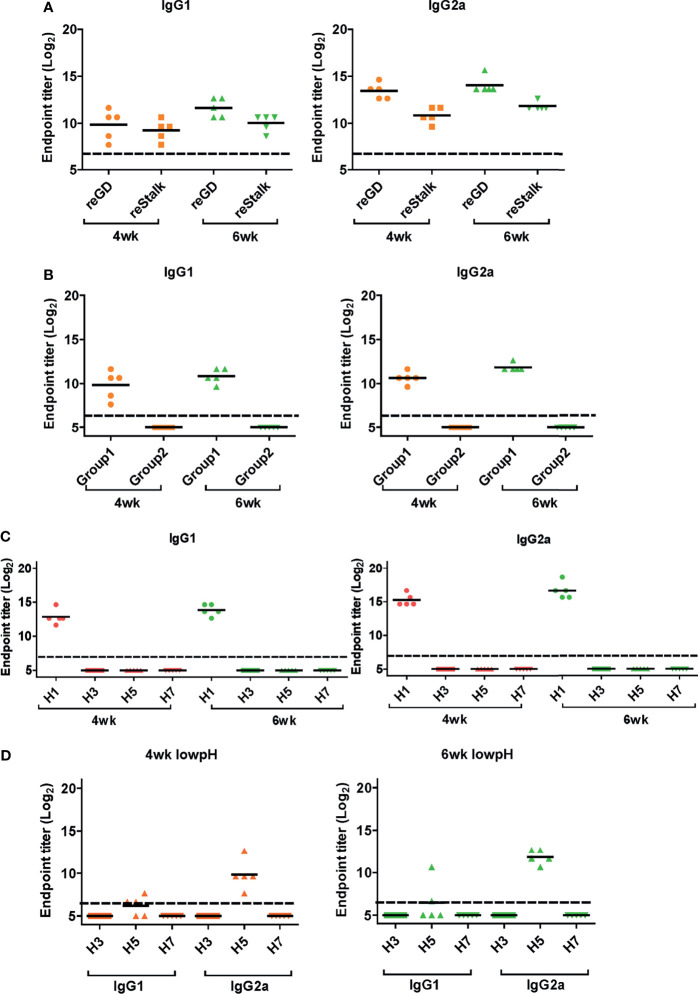
Response of cross-protective antibodies to hemagglutinin (HA) proteins. **(A–D)** Influenza HA protein-specific IgG1 and IgG2a were detected by ELISA analysis. Two-fold serially diluted mice sera collected at 4 or 6 weeks post-vaccination were bound to recombinant GD and HA stalk **(A)**, consensus HA stalk **(B)** expressed in *Escherichia coli*, and commercial (Sino Biological) full HA proteins **(C, D)**. Especially **(D)** HA proteins were pre-treated at low pH to expose the stalk. The graph shows the endpoint (E.P) of each cohort *(N=5).* E.P means the reciprocal serum dilution of the vaccinated group that yielded OD_450_ greater than the mean + 2SD of the PBS-treated group.

**Figure 6:**
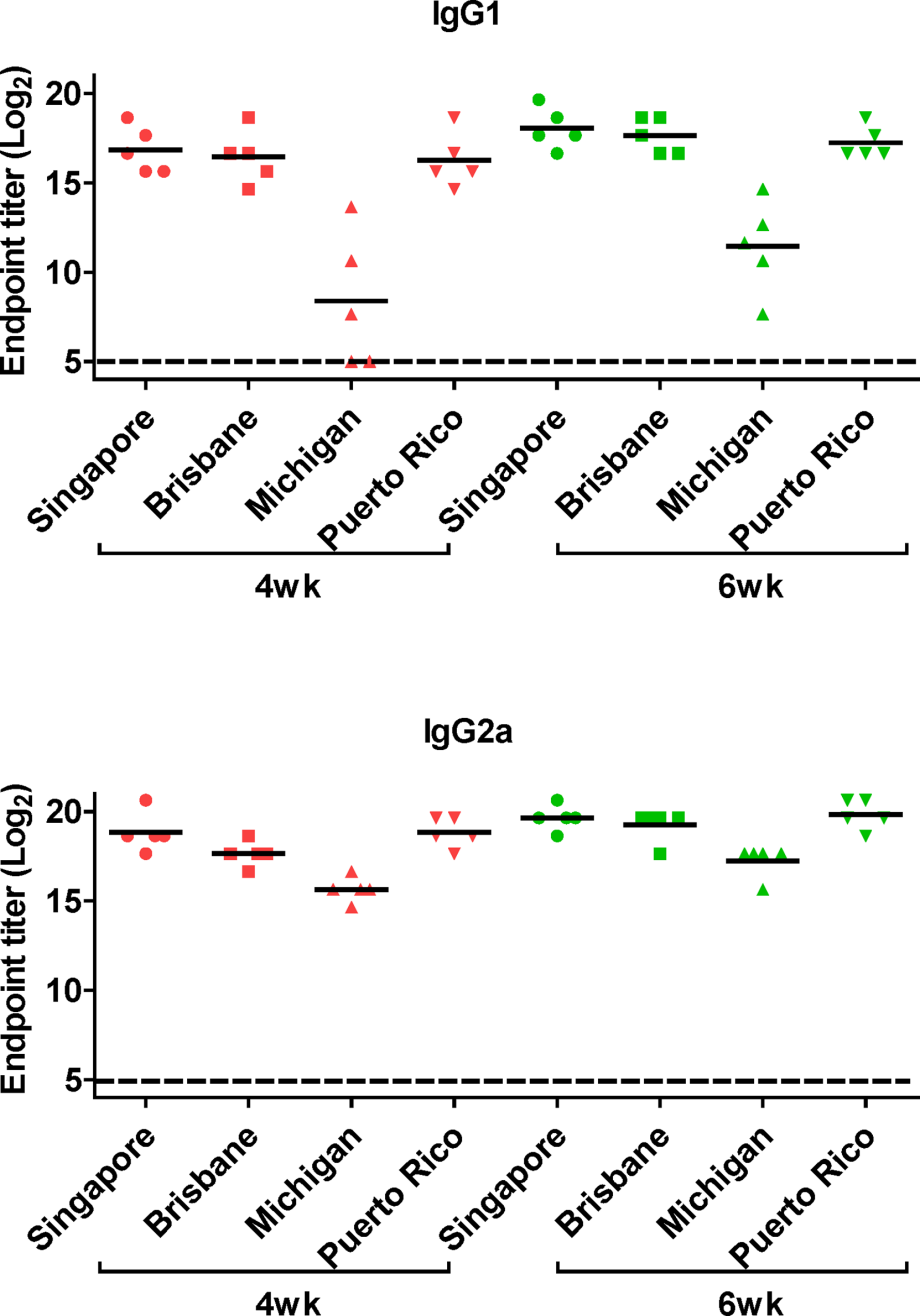
Generation of cross-protective reactive antibodies against the whole hemagglutinin (HA) protein of recombinant H1 strain. HA proteins of H1 strain-specific IgG1 and IgG2a were detected ELISA. Two-fold serial diluted mice sera collected at 4 or 6 weeks posy-vaccination were bound to HA proteins of A/Singapore/Gp1908/15, A/Brisbane/59/07, A/Michigan/45/15, and A/Puerto Rico/8/34. The graph shows the endpoint (E.P) of each cohort *(N=5).* E.P means the reciprocal serum dilution of the vaccinated group that yielded OD_450_ greater than the mean + 2SD of the PBS-treated group.

**Figure 7:**
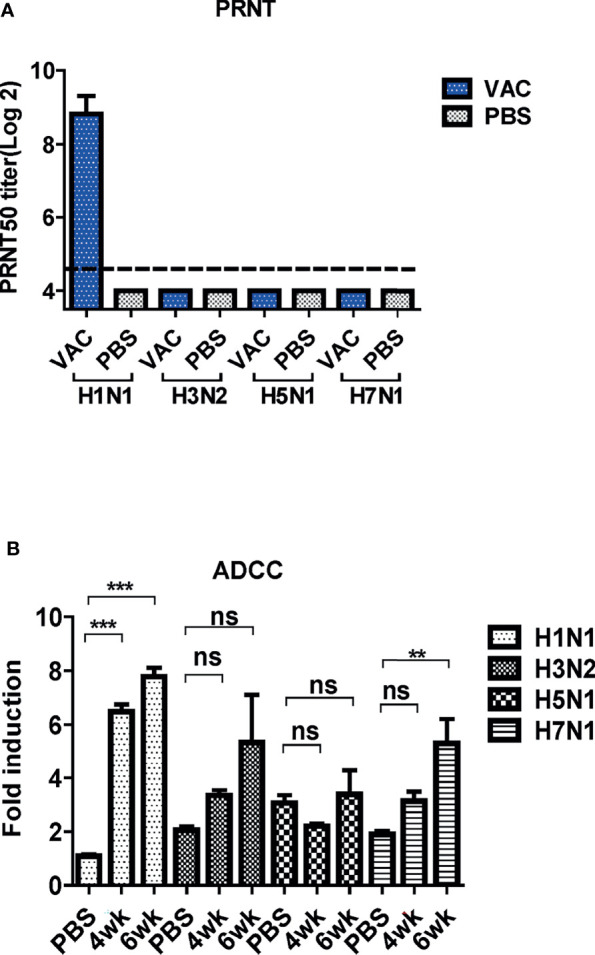
Cross-protective mechanism of immune sera from mice. **(A)** Neutralizing activity of antibody-induced vaccination. Four types of viruses (H1N1, H3N2, H5N1, and H7N1) and mice sera collected 6 weeks post-vaccination were used for determining the neutralizing activity. The detection limit was indicated by a dotted line in the graph. The experiment was repeated twice with the pooled sera of each group *(N=2)*, and the error bar indicates the standard deviation (SD). **(B)** Antibody-dependent cellular cytotoxicity (ADCC) activity of mice sera. ADCC assay was performed by mFcγRIV ADCC reporter bioassay, and fold induction means the RLU (induced – background)/RLU (no Ab control – background). The graph shows the mean of each cohort *(N=5)*, and the error bar means the SD. One-way *ANOVA* test was performed for comparing the differences between sera from 4-week, 6-week, and PBS groups (***P < 0.001; **P < 0.01; ns means not significant).

Next, we performed ELISA to evaluate the cross-reactive serum antibodies induced by DM-C vaccination. The sera obtained at 4 and 6 weeks after vaccination showed significantly higher titers of IgG1 and IgG2a antibodies than those of the PBS group (**Figure 4B**). A high IgG2a titer (with the potential exception of H7) against group 1 and 2 viruses may account for the wide-spectrum ADCC activity (see **Figure 7B** below). When ELISA was performed using recombinant HA globular domain (reGD) and stalk domain (reStalk) of H1N1 (PR8) as coated antigen (27), high titers of IgG1 and IgG2a antibodies were detected at both weeks 4 and 6 (**Figure 5A**). Interestingly, noticeable levels of antibodies against the stalk region were detected, which can act as a promising strategy for conferring broad protection (14, 16). To further investigate stalk-reactive antibodies, ELISA was performed using a consensus HA stalk (cHA) of groups 1 and 2, respectively, which was designed *in silico* as the most conserved part of the HA stalk (**Figure 5B**) (31). The quality of the recombinant HA group-specific stalks was verified previously when they were used for generating HA group-specific mAbs for a new ELISA-based potency assay of an influenza vaccine (28, 31). The serum antibodies induced by DM-C reacted with cHA of group 1 only, but not with cHA of group 2, suggesting that antibodies induced by DM-C do not recognize the HA stalk from different HA groups. Furthermore, sera antibodies elicited by DM-C reacted with only the homologous PR8 HA protein, but not with HA proteins of heterologous origin (H3, H5, and H7) (**Figure 5C**). The results of ELISA with whole HA are in agreement with those of ELISA with the HA stalk (**Figure 5B**), but apparently contradictory for H5 of group 1. To further verify whether the sera antibodies bind to HA stalks of heterologous subtypes, HA proteins of H3, H5, and H7 were pretreated at low pH to expose their stalk regions (31–34). The results showed that stalk-reactive antibodies against group 1 (H5) stalk were present in immune sera from DM-C-vaccinated mice (**Figure 5D**). It showed that immune sera became reactive to the exposed HA stalk of the H5 subtype (same group as H1) by a pH-dependent conformational change of HA (34). Interestingly, a much stronger response was observed with IgG2a than with IgG1 subtype antibodies (**Figure 5D**), which may correlate with the Th1 type-dependent broad-spectrum ADCC activity (see **Figure 7B** below). Since DM-C showed strong systemic antibody responses against the homologous PR8, ELISA was performed against various strains belonging to the H1N1 subtype. Except for the A/Michigan/45/15 strain, most of the strains tested were reactive to IgG1. Interestingly, IgG2a antibodies were found to be cross-reactive with all strains tested (**Figure 6**), probably contributing to effector function-mediated broad-protection (**Figure 7B**). These results suggest that DM-C vaccination effectively induces the production of antibodies that confer cross-protection within the same HA group.

### Antibody-Dependent Protection Mechanisms

The PRNT was performed to examine the cross-neutralization activities of DM-C against the four influenza viruses. The sera antibodies showed potent neutralizing activity against H1N1 (PR8), but not against the other strains (**Figure 7A**). Several studies have shown that LAIVs provide cross-protection even in the absence of neutralizing antibodies (27, 35). Additionally, recent studies have shown that non-neutralizing antibodies induce cross-protection *via* antibody effector functions, such as ADCC (36). Thus, the ADCC bioassay was performed to examine whether cross-reactive sera antibodies induced by DM-C demonstrate ADCC activity in virus-infected cells (37–39). The sera antibodies showed detectable ADCC activity against the virus-infected cells, although the difference was not statistically significant compared to the PBS group (**Figure 7B**). These results suggest that although the antibodies induced by DM-C did not neutralize the heterologous influenza viruses, the effector function, probably mediated by the IgG2a immunoglobulin subtype, exert cross-protective ADCC activities against heterologous influenza viruses.

### Cell-Mediated Immune Responses Elicited by DM-C

**Figure 8:**
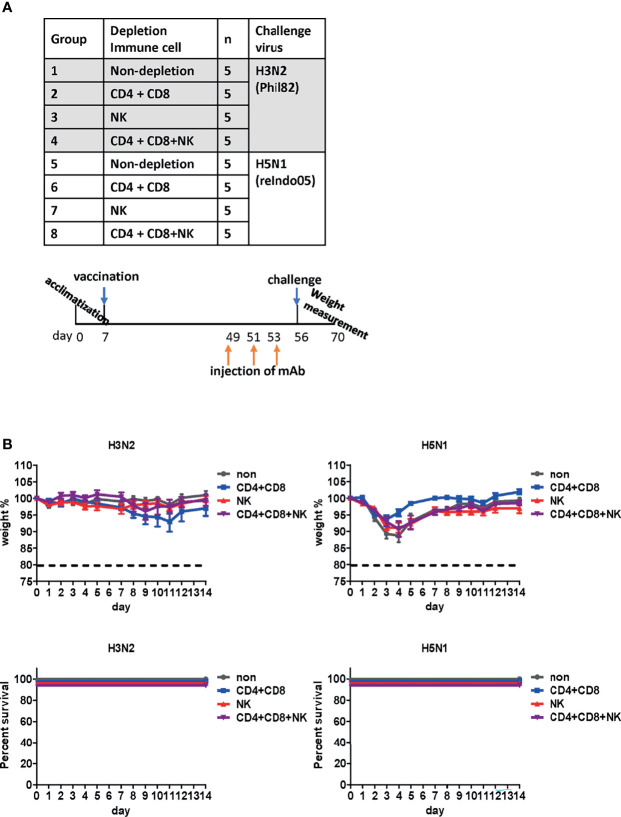
Effect of immune cells against cross-protection in mice. **(A)** Schematic diagram of the schedule of immune cell depletion in mice. To deplete CD4+, CD8+ T cells, and natural killer (NK) cells, mice were injected with corresponding monoclonal antibodies three times on days 1, 3, and 5 before challenge. Immune cell-depleted mice were challenged with H3N2 of group 2 and H5N1 of group 1 viruses. **(B)** Contribution of immune cells towards cross-protection. CD4+, CD8+ T cells, and NK cells were depleted in mice. The body weight of mice was measured daily, and changes in the body weight and survival rate are shown. The humane endpoint is a weight-loss reduction of more than 20%, which is indicated by a dotted line in the graph. The graph shows the mean of each cohort *(N=5)*, and the error bar indicates the standard deviation (SD).

T cells and NK cells have been reported to be critical for cross-protection against influenza viruses (40, 41). To examine their potential contribution, T cells or NK cells were depleted from vaccinated mice before the lethal challenge (**Figure 8A**) by the injection of anti-CD4, anti-CD8, or anti-asialo GM1 antibodies into mice (27). The process depletes CD4+ and CD8+ cells in the peripheral blood and the lung effectively, and partially depletes NK cells in the spleen (27). When challenged with H3N2 belonging to group 2, no notable differences were observed in body weight or mortality. Similarly, we failed to observe any noticeable differences in mice after the H5N1 challenge (**Figure 8B**). Overall, the depletion of CD4+ T cells, CD8+ T cells, and NK cells did not abrogate cross-protection against heterologous virus challenge. These results suggest that antibody-mediated effector function significantly contributes to cross-protection conferred by the DM-C LAIV.

## Discussion

The need for UIVs has been highlighted due to persistent genetic mutations in the influenza virus genome and concerns regarding vaccine mismatches and pandemics. Various approaches have been developed for the production of UIVs, such as recombinant subunit vaccines or DNA vaccines that target highly conserved antigens such as HA stalk or M2e region. A LAIV mimics natural infection and thus provides effective protection by multiple synergistic modes of protection (15, 42). The representative strategy of LAIV is CAIV attenuated by serial passages of the influenza virus at low temperatures (27, 43). Alternatively, attenuation of virulence could be harnessed more precisely using reverse genetic methodology (44–46). We previously constructed a ctLAIV mutant virus, DM-C, that renders self-attenuation through the cleavage of viral NP and NS1 proteins by the host-resident caspase in virus-infected cells (25). NP and NS1 are internal proteins that play pivotal functions during the influenza infection cycle; NP regulates viral replication, whereas NS1 antagonizes the host antiviral response (47, 48). Engineered proteins carrying the caspase-3 and -7 cleavage motif, Asp-Glu-Val-Asp (DEVD) within the structural genes of *NP* and *NS1* (49–52), are subjected to degradation in virus-infected cells, leading to dramatic attenuation of virulence (27). The attenuation properties of DM-C strain were previously characterized in terms of optimal growth temperature, virulence in mice, and the levels of pro-inflammatory cytokines induced by vaccination (25). Here, we showed that a single vaccination with DM-C(group1 H1N1) conferred sterile protection against the group 2 wild type H3N2 viruses in a mouse model (**Figure 2**). The immunization successfully led to complete clearance of the viral load in the respiratory tracts (lungs and nasal turbinates) within 7 dpi (**Figure 3**). The challenge dose 2mLD_50_ was adopted in this experiment. Similarly, sterile protection is achieved from single gene recombinant 7:1 rH5N1 and rH7N1 viruses as well. To be more stringent, higher dose (e.g., 5 or 10 mLD_50)_ could be employed in further studies. The protection is not mediated by HA-specific NT Abs, but probably *via* effector-mediated function (e.g., ADCC) of multiple surface antigens including NA, M2, or even NP (see below) (**Figure 7**). Cross-reactivity of sIgA across group 1 and 2 viruses (with potential exception of H7) may also reflect their contribution to broad protection (**Figure 4A**), especially considering the polymeric nature and proven ability of sIgA for cross-protection (53). Overall, harnessed with the attenuated delivery of whole set of viral antigens, the protection by ctLAIV is mediated by multiple antigens, operated by humoral, mucosal and cellular immune responses.

**Figure 9:**
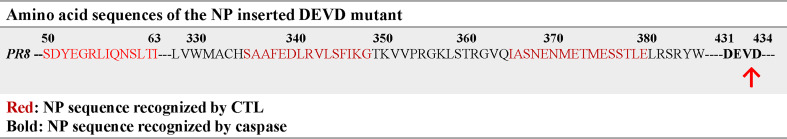
Amino acid sequences of the NP inserted DEVD mutant NP sequences of recognized by CTL were written in red, and NP sequences of recognized by caspase were written in bold. The caspase cleavage site was indicated by red arrow.

Next, we sought to explore the mechanism underlying the observed cross-protection. To address the protective mechanisms, we first analyzed the quality of antibody responses by ELISA. Results of ELISA using whole influenza viruses as coating antigens confirmed strong mucosal response (sIgA in nasal turbinates), and humoral response (IgG1 and IgG2a antibodies in the blood) induced by DM-C, indicating that DM-C probably recognizes multiple surface components of influenza viruses. sIgA antibodies usually form multimers with higher avidity to the antigens than monomeric IgG antibodies, providing on-site protection at the mucosal surface (54, 55). Despite the technical difficulties involved in the collection process of sIgA, our results showed that the IgA subtype was cross-reactive to both group1 and 2 viruses (**Figure 4A**). Therefore, IgA antibodies with high antigen-binding ability may contribute to broad-spectrum cross-protection, as shown in **Figure 2** (53, 56). Whether the low reactivity to H7N1 is related to the immunosuppressive nature of H7 type viruses warrants further investigation (29, 30). The conserved HA stalk region remains a major target in most UIV strategies. Here, we observed that antibodies induced by DM-C recognized the homologous PR8 (H1) strain only, but failed to recognize the HA stalk region of other viruses within the same group 1 (**Figure 5C**). However, the reactivity to H5 (group 1) was greatly increased by low pH treatment, triggering the pH-dependent conformational transition of HA, where the HA stalk became exposed (31–34). These results suggested that antibodies elicited by DM-C could recognize the HA stalk region of the same HA group 1, contributing to cross-protection within group 1 viruses. Inability to bind to the ‘pre-fusion’ conformation of the whole HA at neutral pH (**Figure 5C**) suggests that the protection is not mediated by neutralizing activity before infection, but rather at the later step of infection where ‘post-fusion’ conformation (where the stalk is exposed) becomes manifested. Therefore, antibodies elicited by DM-C vaccination could only neutralize the homologous PR8 virus (**Figure 7A**). Notably, although neutralizing antibodies are considered as the most important immune correlates for strain-specific protection, non-neutralizing antibodies have emerged as important factors especially for broad cross-protection (36, 56). Internal proteins such as NP are highly conserved among viruses and thus are considered important antigens for cross-protection through CTL responses (36, 57). Although the NP of DM-C in host cells is cleaved by caspases (25), NP-specific CTL recognition sites remain unharmed (**Figure 9**) (58, 59), although detailed analyses of CTL responses were beyond the scope of the present study.

Non-neutralizing antibodies have various functions in immune responses, including phagocytosis, complement-dependent cytotoxicity (CDC), and ADCC (36, 60, 61). The Fc gamma receptor of effector cells, such as NK cells, macrophages, and neutrophils, recognize the Fc region of IgG bound to the antigens to mediate the killing of the infected cells (62, 63). As a human counterpart of IgG1 and FcγRIIIa, IgG2a provides its effector function through the murine FcγRIV receptor (64, 65). Using the currently available reporter assay, detectable levels of ADCC antibodies were elicited by DM-C, a prototype H1 type (group 1) vaccine against H3N2 and H7N1 strains (group 2) (**Figure 7B**). Although the ADCC response to H5 is low, the stalk-reactive antibody (**Figure 5D**) may provide an additional layer of protection, probably at the step of viral membrane fusion in the endosome (66), with consequent protection from the lethal challenge (**Figure 2C**). These results suggest that ADCC contributes to DM-C-mediated cross-protection. Although most of our analysis was performed on HA, the possibility that DM-C LAIV vaccination could also elicit immune responses to other surface proteins, such as M2 or neuraminidase (NA), should not be ruled out. Our analysis showed that IgG2a antibodies were also elicited against the M2 protein (**Figure S1** in **Supplementary Material**). M2 is the third most abundant protein after HA and NA among the proteins present on the surface of virions. Since M2 is highly conserved, it is also a target for UIV strategies (37). Rather than directly neutralizing the virus, M2 antibodies play protective roles through antibody effector functions (42, 67, 68). Therefore, DM-C may provide an additional layer of protection by ADCC function targeting both M2 and NA. It was shown that NP, previously recognized as a strictly internal protein, could be displayed on the surface of infected cells (69). However, whether NP is an additional target for ADCC remains to be investigated (39). In addition to IgG, IgA is recognized by the Fc alpha receptor in humans to induce ADCC (70, 71). Unfortunately, it is difficult to experimentally evaluate the ADCC activity of IgA *in vitro* using a mouse model because they lack receptors that play the same role as that of the human macrophage Fc alpha receptor (72).

The depletion of T cells and NK cells did not completely abolish the protection against lethal challenges with heterologous viruses. Cross-protection remained persistent even after depletion of T cells or NK cells, suggesting that antibody-mediated mechanisms are a major arm for cross-protection by DM-C. LAIV is very similar to the wild virus, except for the low virulence level, while delivering all surface and internal proteins. Therefore, DM-C may induce multiple layers of immune responses toward effective protection (42). In this study, DM-C induced IgA mucosal response, as well as IgG1 and IgG2a-type humoral responses, recognizing most of the IAVs tested. Most crucially, DM-C vaccination led to the complete clearance of all types of infecting viruses tested (**Figure 3**), tantamount to pan-influenza A protection. It should be emphasized that all animals in this study were challenged seven weeks after vaccination. Therefore, it could be concluded that cross-protection of DM-C was due to adaptive immunity rather than innate immunity, which usually provides immediate, non-specific protection for only a short period (73, 74).

Our study presents the possibility of a universal vaccine that covers both HA group 1and 2 viruses. A full protection against H3N2 challenge – as reflected in mortality, viral clearance in lung and nasal turbinate - strongly supports cross-protection covering group1 and 2, regardless of the constellation of surface and internal proteins. However, it should be mentioned that, for H5 and H7 challenge, single gene reassortant viruses [sub-lethal in some instance (**Figure 2**)], rather than wild type highly pathogenic avian influenza (HPAI) viruses, were used due to limited access to BSL3 facilities. The limitations in our experimental setting should be dully considered in our interpretation on the breadth of cross-protection especially against avian viruses. Second, establishing the immune correlate of cross (potentially universal) protection remains difficult (10). The issue is even more challenging especially for LAIVs that delivers a variety of viral antigens and provides multiple layer of protection (22, 42, 56). First, the antibody effector function ADCC (**Figure 7B**) could be mediated not only by the classic HA, NA, M2 surface proteins, but by NP as well, previously known as internal proteins, but could also be displayed on the surface of infected cells (26). The relative contributions of each component should be further analyzed preferably by depletion of specific antibodies or by using specific antigen expressing cells in ADCC assay. In addition, further evaluation is required for mucosal secretory IgA responses (in **Figure 4**) as an additional layer of protection especially for LAIVs. Third, the contribution of T cells and NK cells in cross-protection was approached by their depletion by mAbs against cellular markers. Our experience has shown that depletion of CD4+ and CD8+ was effective, but that of NK cells from spleen is not complete (27), leaving the room for further evaluation of the role of ADCC function in cross-protection. Therefore, the present results of potency and breadth of protection addressed in the mouse model by single immunization should be further confirmed and validated in future exploration, preferably in clinically relevant ferret models with wild type challenges.

## Data Availability Statement

The original contributions presented in the study are included in the article/**Supplementary Material**. Further inquiries can be directed to the corresponding authors.

## Ethics Statement

The animal study was reviewed and approved by the Institutional Animal Care and Use Committee (IACUC) of the International Vaccine Institute (IVI).

## Author Contributions

MK, YJ, JiL, and BS designed the study. MK, SB, YJ, and BS analyzed the data and wrote the manuscript. MK performed overall experiments. YC and JoL contributed to animal experiments. YJ, SB, and BS supervised all process of experiments and preparation of the manuscript. All authors contributed to the article and approved the submitted version.

## Funding

This work was supported by a research grant funded by the Andong-type Job Project Group in Andong National University.

## Conflict of Interest

The authors declare that the research was conducted in the absence of any commercial or financial relationships that could be construed as a potential conflict of interest.

## Publisher’s Note

All claims expressed in this article are solely those of the authors and do not necessarily represent those of their affiliated organizations, or those of the publisher, the editors and the reviewers. Any product that may be evaluated in this article, or claim that may be made by its manufacturer, is not guaranteed or endorsed by the publisher.

## References

[1] Iuliano AD Roguski KM Chang HH Muscatello DJ Palekar R Tempia S. Estimates of Global Seasonal Influenza-Associated Respiratory Mortality: A Modelling Study. Lancet (2018) 391(10127):1285–300. doi: 10.1016/S0140-6736(17)33293-2 PMC5935243 29248255

[2] Webster RG Bean WJ Gorman OT Chambers TM Kawaoka Y. Evolution and Ecology of Influenza A Viruses. Microbiol Rev (1992) 56(1):152–79. doi: 10.1128/mr.56.1.152-179.1992 PMC372859 1579108

[3] Hampson AW Mackenzie JS. The Influenza Viruses. Med J Aust (2006) 185(S10):S39–43. doi: 10.5694/j.1326-5377.2006.tb00705.x 17115950

[4] Carrat F Flahault A. Influenza Vaccine: The Challenge of Antigenic Drift. Vaccine (2007) 25(39-40):6852–62. doi: 10.1016/j.vaccine.2007.07.027 17719149

[5] Treanor J. Influenza Vaccine–Outmaneuvering Antigenic Shift and Drift. N Engl J Med (2004) 350(3):218–20. doi: 10.1056/NEJMp038238 14724300

[6] Gerdil C. The Annual Production Cycle for Influenza Vaccine. Vaccine (2003) 21(16):1776–9. doi: 10.1016/s0264-410x(03)00071-9 12686093

[7] Tricco AC Chit A Soobiah C Hallett D Meier G Chen MH. Comparing Influenza Vaccine Efficacy Against Mismatched and Matched Strains: A Systematic Review and Meta-Analysis. BMC Med (2013) 11:153. doi: 10.1186/1741-7015-11-153 23800265 PMC3706345

[8] Palese P. Influenza: Old and New Threats. Nat Med (2004) 10(12 Suppl):S82–7. doi: 10.1038/nm1141 15577936

[9] Horimoto T Kawaoka Y. Influenza: Lessons From Past Pandemics, Warnings From Current Incidents. Nat Rev Microbiol (2005) 3(8):591–600. doi: 10.1038/nrmicro1208 16064053

[10] Erbelding EJ Post DJ Stemmy EJ Roberts PC Augustine AD Ferguson S. A Universal Influenza Vaccine: The Strategic Plan for the National Institute of Allergy and Infectious Diseases. J Infect Dis (2018) 218(3):347–54. doi: 10.1093/infdis/jiy103 PMC6279170 29506129

[11] Krammer F Garcia-Sastre A Palese P. Is It Possible to Develop a "Universal" Influenza Virus Vaccine? Potential Target Antigens and Critical Aspects for a Universal Influenza Vaccine. Cold Spring Harb Perspect Biol (2018) 10(7):a028845. doi: 10.1101/cshperspect.a028845 28663209 PMC6028071

[12] Coughlan L Palese P. Overcoming Barriers in the Path to a Universal Influenza Virus Vaccine. Cell Host Microbe (2018) 24(1):18–24. doi: 10.1016/j.chom.2018.06.016 30001520

[13] Kumar A Meldgaard TS Bertholet S. Novel Platforms for the Development of a Universal Influenza Vaccine. Front Immunol (2018) 9:600. doi: 10.3389/fimmu.2018.00600 29628926 PMC5877485

[14] Hai R Krammer F Tan GS Pica N Eggink D Maamary J. Influenza Viruses Expressing Chimeric Hemagglutinins: Globular Head and Stalk Domains Derived From Different Subtypes. J Virol (2012) 86(10):5774–81. doi: 10.1128/JVI.00137-12 PMC3347257 22398287

[15] Jang YH Seong BL. The Quest for a Truly Universal Influenza Vaccine. Front Cell Infect Microbiol (2019) 9:344. doi: 10.3389/fcimb.2019.00344 31649895 PMC6795694

[16] Krammer F Palese P Steel J. Advances in Universal Influenza Virus Vaccine Design and Antibody Mediated Therapies Based on Conserved Regions of the Hemagglutinin. Curr Top Microbiol Immunol (2015) 386:301–21. doi: 10.1007/82_2014_408 25007847

[17] Schotsaert M De Filette M Fiers W Saelens X. Universal M2 Ectodomain-Based Influenza A Vaccines: Preclinical and Clinical Developments. Expert Rev Vaccines (2009) 8(4):499–508. doi: 10.1586/erv.09.6 19348565 PMC2706389

[18] Nachbagauer R Liu WC Choi A Wohlbold TJ Atlas T Rajendran M. A Universal Influenza Virus Vaccine Candidate Confers Protection Against Pandemic H1N1 Infection in Preclinical Ferret Studies. NPJ Vaccines (2017) 2:26. doi: 10.1038/s41541-017-0026-4 29263881 PMC5627297

[19] Jang YH Seong BL. Options and Obstacles for Designing a Universal Influenza Vaccine. Viruses (2014) 6(8):3159–80. doi: 10.3390/v6083159 PMC4147691 25196381

[20] Krammer F. The Human Antibody Response to Influenza A Virus Infection and Vaccination. Nat Rev Immunol (2019) 19(6):383–97. doi: 10.1038/s41577-019-0143-6 30837674

[21] Zhou B Meliopoulos VA Wang W Lin X Stucker KM Halpin RA. Reversion of Cold-Adapted Live Attenuated Influenza Vaccine Into a Pathogenic Virus. J Virol (2016) 90(19):8454–63. doi: 10.1128/JVI.00163-16 PMC5021423 27440882

[22] Jang YH Seong BL. Principles Underlying Rational Design of Live Attenuated Influenza Vaccines. Clin Exp Vaccine Res (2012) 1(1):35–49. doi: 10.7774/cevr.2012.1.1.35 23596576 PMC3623510

[23] Labbe K Saleh M. Cell Death in the Host Response to Infection. Cell Death Differ (2008) 15(9):1339–49. doi: 10.1038/cdd.2008.91 18566602

[24] Barber GN. Host Defense, Viruses and Apoptosis. Cell Death Differ (2001) 8(2):113–26. doi: 10.1038/sj.cdd.4400823 11313713

[25] Jang YH Byun YH Lee KH Park ES Lee YH Lee YJ. Host Defense Mechanism-Based Rational Design of Live Vaccine. PloS One (2013) 8(10):e75043. doi: 10.1371/journal.pone.0075043 24098364 PMC3788757

[26] Von Holle TA Moody MA. Influenza and Antibody-Dependent Cellular Cytotoxicity. Front Immunol (2019) 10:1457. doi: 10.3389/fimmu.2019.01457 31316510 PMC6611398

[27] Jang YH Kim JY Byun YH Son A Lee JY Lee YJ. Pan-Influenza A Protection by Prime-Boost Vaccination With Cold-Adapted Live-Attenuated Influenza Vaccine in a Mouse Model. Front Immunol (2018) 9:116. doi: 10.3389/fimmu.2018.00116 29449842 PMC5799225

[28] Chae W Kim P Kim H Cheong YC Kim YS Kang SM. Hemagglutinin Quantitative ELISA-Based Potency Assay for Trivalent Seasonal Influenza Vaccine Using Group-Specific Universal Monoclonal Antibodies. Sci Rep (2019) 9(1):19675. doi: 10.1038/s41598-019-56169-5 31873147 PMC6927952

[29] Schmeisser F Vasudevan A Verma S Wang W Alvarado E Weiss C. Antibodies to Antigenic Site A of Influenza H7 Hemagglutinin Provide Protection Against H7N9 Challenge. PloS One (2015) 10(1):e0117108. doi: 10.1371/journal.pone.0117108 25629172 PMC4309539

[30] Guo L Zhang X Ren L Yu X Chen L Zhou H. Human Antibody Responses to Avian Influenza A(H7N9) Virus, 2013. Emerg Infect Dis (2014) 20(2):192–200. doi: 10.3201/eid2002.131094 24447423 PMC3901473

[31] Chae W Kim P Hwang BJ Seong BL. Universal Monoclonal Antibody-Based Influenza Hemagglutinin Quantitative Enzyme-Linked Immunosorbent Assay. Vaccine (2019) 37(11):1457–66. doi: 10.1016/j.vaccine.2019.01.068 30765169

[32] White JM Wilson IA. Anti-Peptide Antibodies Detect Steps in a Protein Conformational Change: low-pH Activation of the Influenza Virus Hemagglutinin. J Cell Biol (1987) 105(6 Pt 2):2887–96. doi: 10.1083/jcb.105.6.2887 PMC2114698 2447101

[33] Harrison SC. Viral Membrane Fusion. Nat Struct Mol Biol (2008) 15(7):690–8. doi: 10.1038/nsmb.1456 PMC2517140 18596815

[34] Carr CM Kim PS. A Spring-Loaded Mechanism for the Conformational Change of Influenza Hemagglutinin. Cell (1993) 73(4):823–32. doi: 10.1016/0092-8674(93)90260-w 8500173

[35] Powell TJ Strutt T Reome J Hollenbaugh JA Roberts AD Woodland DL. Priming With Cold-Adapted Influenza A Does Not Prevent Infection But Elicits Long-Lived Protection Against Supralethal Challenge With Heterosubtypic Virus. J Immunol (2007) 178(2):1030–8. doi: 10.4049/jimmunol.178.2.1030 17202366

[36] Jegaskanda S Reading PC Kent SJ. Influenza-Specific Antibody-Dependent Cellular Cytotoxicity: Toward a Universal Influenza Vaccine. J Immunol (2014) 193(2):469–75. doi: 10.4049/jimmunol.1400432 24994909

[37] Deng L Cho KJ Fiers W Saelens X. M2e-Based Universal Influenza A Vaccines. Vaccines (Basel) (2015) 3(1):105–36. doi: 10.3390/vaccines3010105 PMC4494237 26344949

[38] Tan GS Leon PE Albrecht RA Margine I Hirsh A Bahl J. Broadly-Reactive Neutralizing and Non-Neutralizing Antibodies Directed Against the H7 Influenza Virus Hemagglutinin Reveal Divergent Mechanisms of Protection. PloS Pathog (2016) 12(4):e1005578. doi: 10.1371/journal.ppat.1005578 27081859 PMC4833315

[39] Jegaskanda S Co MDT Cruz J Subbarao K Ennis FA Terajima M. Induction of H7N9-Cross-Reactive Antibody-Dependent Cellular Cytotoxicity Antibodies by Human Seasonal Influenza A Viruses That are Directed Toward the Nucleoprotein. J Infect Dis (2017) 215(5):818–23. doi: 10.1093/infdis/jiw629 PMC5853654 28011910

[40] Clemens EB van de Sandt C Wong SS Wakim LM Valkenburg SA. Harnessing the Power of T Cells: The Promising Hope for a Universal Influenza Vaccine. Vaccines (Basel) (2018) 6(2):18. doi: 10.3390/vaccines6020018 PMC6027237 29587436

[41] Balz K Trassl L Hartel V Nelson PP Skevaki C. Virus-Induced T Cell-Mediated Heterologous Immunity and Vaccine Development. Front Immunol (2020) 11:513. doi: 10.3389/fimmu.2020.00513 32296430 PMC7137989

[42] Jang YH Seong BL. Immune Responses Elicited by Live Attenuated Influenza Vaccines as Correlates of Universal Protection Against Influenza Viruses. Vaccines (Basel) (2021) 9(4):353. doi: 10.3390/vaccines9040353 33916924 PMC8067561

[43] Isakova-Sivak I Chen LM Matsuoka Y Voeten JT Kiseleva I Heldens JG. Genetic Bases of the Temperature-Sensitive Phenotype of a Master Donor Virus Used in Live Attenuated Influenza Vaccines: A/Leningrad/134/17/57 (H2n2). Virology (2011) 412(2):297–305. doi: 10.1016/j.virol.2011.01.004 21315402

[44] Zhu Q Yang H Chen W Cao W Zhong G Jiao P. A Naturally Occurring Deletion in Its NS Gene Contributes to the Attenuation of an H5N1 Swine Influenza Virus in Chickens. J Virol (2008) 82(1):220–8. doi: 10.1128/JVI.00978-07 PMC2224367 17942562

[45] Luytjes W Krystal M Enami M Parvin JD Palese P. Amplification, Expression, and Packaging of Foreign Gene by Influenza Virus. Cell (1989) 59(6):1107–13. doi: 10.1016/0092-8674(89)90766-6 2598262

[46] Palese P Zheng H Engelhardt OG Pleschka S Garcia-Sastre A. Negative-Strand RNA Viruses: Genetic Engineering and Applications. Proc Natl Acad Sci U.S.A. (1996) 93(21):11354–8. doi: 10.1073/pnas.93.21.11354 PMC38061 8876139

[47] Liao TL Wu CY Su WC Jeng KS Lai MM. Ubiquitination and Deubiquitination of NP Protein Regulates Influenza A Virus RNA Replication. EMBO J (2010) 29(22):3879–90. doi: 10.1038/emboj.2010.250 PMC2989104 20924359

[48] Hale BG Randall RE Ortin J Jackson D. The Multifunctional NS1 Protein of Influenza A Viruses. J Gen Virol (2008) 89(Pt 10):2359–76. doi: 10.1099/vir.0.2008/004606-0 18796704

[49] Thornberry NA Rano TA Peterson EP Rasper DM Timkey T Garcia-Calvo M. A Combinatorial Approach Defines Specificities of Members of the Caspase Family and Granzyme B. Functional Relationships Established for Key Mediators of Apoptosis. J Biol Chem (1997) 272(29):17907–11. doi: 10.1074/jbc.272.29.17907 9218414

[50] Nicholson DW. Caspase Structure, Proteolytic Substrates, and Function During Apoptotic Cell Death. Cell Death Differ (1999) 6(11):1028–42. doi: 10.1038/sj.cdd.4400598 10578171

[51] Talanian RV Quinlan C Trautz S Hackett MC Mankovich JA Banach D. Substrate Specificities of Caspase Family Proteases. J Biol Chem (1997) 272(15):9677–82. doi: 10.1074/jbc.272.15.9677 9092497

[52] Roulston A Marcellus RC Branton PE. Viruses and Apoptosis. Annu Rev Microbiol (1999) 53:577–628. doi: 10.1146/annurev.micro.53.1.577 10547702

[53] Asahi-Ozaki Y Yoshikawa T Iwakura Y Suzuki Y Tamura S Kurata T. Secretory IgA Antibodies Provide Cross-Protection Against Infection With Different Strains of Influenza B Virus. J Med Virol (2004) 74(2):328–35. doi: 10.1002/jmv.20173 15332283

[54] Suzuki T Kawaguchi A Ainai A Tamura S Ito R Multihartina P. Relationship of the Quaternary Structure of Human Secretory IgA to Neutralization of Influenza Virus. Proc Natl Acad Sci USA (2015) 112(25):7809–14. doi: 10.1073/pnas.1503885112 PMC4485102 26056267

[55] Muramatsu M Yoshida R Yokoyama A Miyamoto H Kajihara M Maruyama J. Comparison of Antiviral Activity Between IgA and IgG Specific to Influenza Virus Hemagglutinin: Increased Potential of IgA for Heterosubtypic Immunity. PloS One (2014) 9(1):e85582. doi: 10.1371/journal.pone.0085582 24465606 PMC3895000

[56] Jang YH Seong BL. Cross-Protective Immune Responses Elicited by Live Attenuated Influenza Vaccines. Yonsei Med J (2013) 54(2):271–82. doi: 10.3349/ymj.2013.54.2.271 PMC3575970 23364956

[57] Zheng M Luo J Chen Z. Development of Universal Influenza Vaccines Based on Influenza Virus M and NP Genes. Infection (2014) 42(2):251–62. doi: 10.1007/s15010-013-0546-4 24178189

[58] Bastin J Rothbard J Davey J Jones I Townsend A. Use of Synthetic Peptides of Influenza Nucleoprotein to Define Epitopes Recognized by Class I-Restricted Cytotoxic T Lymphocytes. J Exp Med (1987) 165(6):1508–23. doi: 10.1084/jem.165.6.1508 PMC2188365 2438367

[59] Townsend AR Rothbard J Gotch FM Bahadur G Wraith D McMichael AJ. The Epitopes of Influenza Nucleoprotein Recognized by Cytotoxic T Lymphocytes can be Defined With Short Synthetic Peptides. Cell (1986) 44(6):959–68. doi: 10.1016/0092-8674(86)90019-x 2420472

[60] Huber VC Lynch JM Bucher DJ Le J Metzger DW. Fc Receptor-Mediated Phagocytosis Makes a Significant Contribution to Clearance of Influenza Virus Infections. J Immunol (2001) 166(12):7381–8. doi: 10.4049/jimmunol.166.12.7381 11390489

[61] Greenberg SB Criswell BS Six HR Couch RB. Lymphocyte Cytotoxicity to Influenza Virus-Infected Cells: Response to Vaccination and Virus Infection. Infect Immun (1978) 20(3):640–5. doi: 10.1128/iai.20.3.640-645.1978 PMC421906 669816

[62] Mellman I Koch T Healey G Hunziker W Lewis V Plutner H. Structure and Function of Fc Receptors on Macrophages and Lymphocytes. J Cell Sci Suppl (1988) 9:45–65. doi: 10.1242/jcs.1988.supplement_9.3 2978517

[63] Tamm A Schmidt RE. IgG Binding Sites on Human Fc Gamma Receptors. Int Rev Immunol (1997) 16(1-2):57–85. doi: 10.3109/08830189709045703 9651786

[64] Nimmerjahn F Bruhns P Horiuchi K Ravetch JV. FcgammaRIV: A Novel FcR With Distinct IgG Subclass Specificity. Immunity (2005) 23(1):41–51. doi: 10.1016/j.immuni.2005.05.010 16039578

[65] Nimmerjahn F Lux A Albert H Woigk M Lehmann C Dudziak D. FcgammaRIV Deletion Reveals Its Central Role for IgG2a and IgG2b Activity In Vivo. Proc Natl Acad Sci USA (2010) 107(45):19396–401. doi: 10.1073/pnas.1014515107 PMC2984189 20974962

[66] Hamilton BS Whittaker GR Daniel S. Influenza Virus-Mediated Membrane Fusion: Determinants of Hemagglutinin Fusogenic Activity and Experimental Approaches for Assessing Virus Fusion. Viruses (2012) 4(7):1144–68. doi: 10.3390/v4071144 PMC3407899 22852045

[67] Fu TM Freed DC Horton MS Fan J Citron MP Joyce JG. Characterizations of Four Monoclonal Antibodies Against M2 Protein Ectodomain of Influenza A Virus. Virology (2009) 385(1):218–26. doi: 10.1016/j.virol.2008.11.035 19070878

[68] Fiers W De Filette M El Bakkouri K Schepens B Roose K Schotsaert M. M2e-Based Universal Influenza A Vaccine. Vaccine (2009) 27(45):6280–3. doi: 10.1016/j.vaccine.2009.07.007 19840661

[69] Virelizier JL Allison AC Oxford JS Schild GC. Early Presence of Ribonucleoprotein Antigen on Surface of Influenza Virus-Infected Cells. Nature (1977) 266(5597):52–4. doi: 10.1038/266052a0 840296

[70] Zwick A Bernhard M Knoerck A Linxweiler M Schick B Heinzelmann J. Monitoring Kinetics Reveals Critical Parameters of IgA-Dependent Granulocyte-Mediated Anti-Tumor Cell Cytotoxicity. J Immunol Methods (2019) 473:112644. doi: 10.1016/j.jim.2019.112644 31404549

[71] Otten MA van Egmond M. The Fc Receptor for IgA (FcalphaRI, Cd89). Immunol Lett (2004) 92(1-2):23–31. doi: 10.1016/j.imlet.2003.11.018 15081523

[72] Reljic R. In Search of the Elusive Mouse Macrophage Fc-Alpha Receptor. Immunol Lett (2006) 107(1):80–1. doi: 10.1016/j.imlet.2006.04.014 16837064

[73] Seo SU Lee KH Byun YH Kweon MN Seong BL. Immediate and Broad-Spectrum Protection Against Heterologous and Heterotypic Lethal Challenge in Mice by Live Influenza Vaccine. Vaccine (2007) 25(47):8067–76. doi: 10.1016/j.vaccine.2007.09.012 17919786

[74] Lee YJ Lee JY Jang YH Seo SU Chang J Seong BL. Non-Specific Effect of Vaccines: Immediate Protection Against Respiratory Syncytial Virus Infection by a Live Attenuated Influenza Vaccine. Front Microbiol (2018) 9:83. doi: 10.3389/fmicb.2018.00083 29445364 PMC5797773

