# Supplementary material for: A Host-Restricted Self-Attenuated Influenza Virus Provides Broad Pan-Influenza A Protection in a Mouse Model
Source: Front Immunol. 2021 Dec 2;12:779223. doi: 10.3389/fimmu.2021.779223 (PMC8674563; doi:10.3389/fimmu.2021.779223)
Supplement: Supplementary file 1 [file DataSheet_1.docx]

Supplementary Material

# Supplementary Data

## Supplementary Figures

**
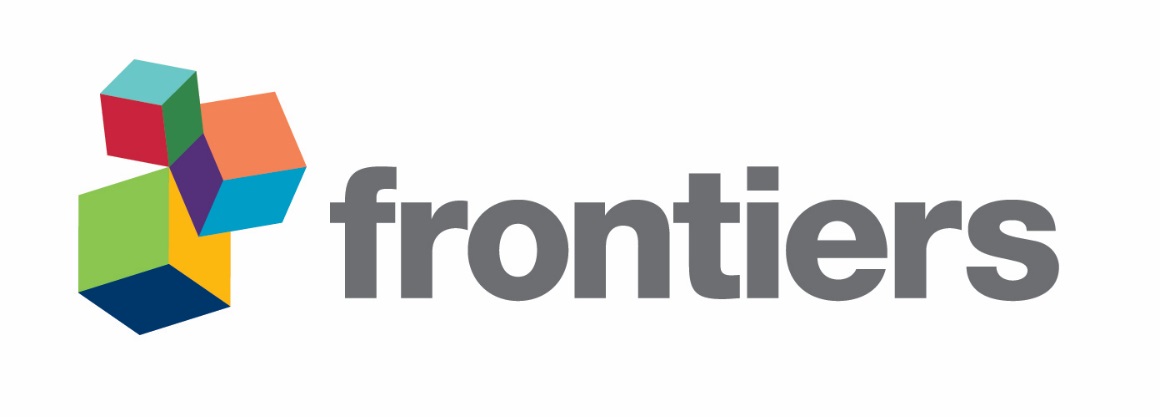
**

**Supplementary Figure 1. Antibody response to M2 protein.**

M2 protein-specific IgG2a antibodies were detected by ELISA analysis. Two-fold serial diluted sera collected from mice at 4 or 6 weeks post-vaccination were bound to M2 protein expressed in *Escherichia coli.*

.
